# Oral Problems in Oncology Patients Undergoing Chemotherapy for Solid Tumors: A Prospective Observational Study

**DOI:** 10.3390/cancers16010176

**Published:** 2023-12-29

**Authors:** Giulia Ottaviani, Giada Targato, Katia Rupel, Margherita Gobbo, Daniele Generali, Alessandra Guglielmi, Angela Dicorato, Daniela Adamo, Federica Canfora, Roberto Di Lenarda, Matteo Biasotto

**Affiliations:** 1 Department of Medical, Surgical and Health Sciences, University of Trieste, Strada di Fiume 447, 34149 Trieste, Italy; 2 Department od Medicine (DAME), University of Udine, 33100 Udine, Italy; 3 Department of Oncology, Udine Academic Hospital, Azienda Sanitaria Universitaria Friuli Centrale (ASUFC), 33100 Udine, Italy; 4 Unit of Oral and Maxillofacial Surgery, Ca’ Foncello Hospital, 31100 Treviso, Italy; 5 Department of Oncology, Maggiore Hospital, Piazza dell’Ospitale 1, 34125 Trieste, Italy; 6 Department of Neuroscience, Reproductive Sciences and Dentistry, University of Naples Federico II, 5 Via Pansini, 80131 Naples, Italy

**Keywords:** chemotherapy, solid tumors, oral problems, stomatotoxicity, numb chin syndrome

## Abstract

**Simple Summary:**

Several oral problems occurring during cancer treatment can lead to the discontinuation or interruption of the scheduled treatment, with a negative impact on patient’s overall survival. Very few studies focused on patients’ self-reported oral problems during chemotherapy treatment for solid tumors. Through the administration of a dedicated questionnaire, we aim at correlating the presence of oral complications to demographic and medical information. Metastatic disease represented a risk factor for the onset of oral mucositis and salivary gland hypofunction, while specific chemotherapy regimens increased the risk to develop a subjective reduction in the salivary flow and difficulty in swallowing. Most of the participants were informed by the oncologist about the possibility of oral problems arising during oncological therapies. It is of paramount importance to collect observational data on oral problems from the patients’ perspective in order to plan information and prevention campaigns to inform patients about their possible occurrence by providing useful tools for prevention and management.

**Abstract:**

PURPOSE: Oral problems in a group of oncological patients undergoing chemotherapy (CT) for solid tumors have been examined. Incidence and severity of patients’ self-reported oral problems have been evaluated along their interaction with age, gender, tumor diagnosis and stage, presence of mestastasis, CT agent type, and number of CT cycle. We also analyzed the presence of paraesthesia and anaesthesia and their predisposing factors associated with clinical and treatment-related variables. METHODS: Patients were asked to fill in a questionnaire to evaluate the onset and the intensity of oral and perioral pain, oral mucositis, salivary gland hypofunction, dysgeusia, dysphagia, dysphonia, and sensitivity neuropathy (paraesthesia or dysaesthesia) since the last CT infusion. We also investigated which types of medications have possibly been used and who recommended it, as well as patients’ degree of awareness about the possibility of oral problems arising during CT. RESULTS: We recruited 194 patients and obtained 491 questionnaires. We found that a metastatic disease was a risk factor for OM (OR 2.02, *p* = 0.026) and salivary gland hypofunction (OR 1.66, *p* = 0.042) and that platinum agents, compared to mitotic inhibitors, increased the risk of developing salivary gland hypofunction (OR 2.16, *p* = 0.013), dysphagia (OR 3.26, *p* = 0.001), and anaesthesia (OR 5.16, *p* = 0.041). Young age was a slight protective factor for most symptoms. The 80% of enrolled patients were informed by the oncologist about possible oral problems arising during CT. CONCLUSIONS: Our study highlighted the importance of collecting observational data from the patients’ perspective on oral problems arising during the routine oncology practice, across a range of solid tumors and CT regimens. The relevance of these findings focused on the key role of the multidisciplinary team in advising the patients on the possible occurrence of oral problems, also by recommending their management.

## 1. Introduction

Patients with solid tumors may suffer from oral problems during their oncological treatment. The great part of these disorders is due to drug stomatotoxicity, but they could also be related to the tumor progression itself. Despite the advances in cancer treatment, chemotherapy (CT) is still widely used alone or in combination with surgery, radiotherapy (RT), targeted therapy, hormone therapy, or immunotherapy. The main side effect of CT is the lack of selectivity, inducing the damage not only for the neoplasm but also for the healthy cells characterized by high proliferation. With specific regard to the oral cavity mucosal cells [1], according to the MASCC/ISOO evidence-based guidance [2], oral problems in patients with cancer can manifest, among others, as oral pain and/or oral mucositis (OM) often associated with oral infections with possible systemic dissemination, salivary gland hypofunction, taste disturbance (dysgeusia), dysphagia, and difficulty in speaking (dysphonia) [3,4,5].

OM implies an inflammation of the oral mucosa with an estimated incidence of 40–50% in patients receiving CT and in almost all patients (80–100%) undergoing head and neck RT [6]. Usually, the OM presents with oedema and erythema associated with pain and a burning sensation with potential difficulty in swallowing. In addition, the interruption of the continuity of the mucosal barrier and the CT-induced neutropenia can increase the risk of local infections or, rarely but dramatically, of systemic spread [7].

The stomatotoxic effect of CT may also involve the major and minor salivary glands, compromising the quality and quantity of saliva. Hyposalivation may occur following CT, radioactive iodine treatment, hematopoietic stem cell transplantation (HSCT), head and neck RT, targeted therapy, and immunotherapy. Moreover, it may be related to medications used to support the cancer patient (e.g., opioid analgesics, centrally acting pain medications) and with dehydration [8]. Hyposalivation leads to a reduction in mucosal protective factors and antimicrobial substances contained in saliva, with consequent increased risk of oral infections and tooth decay [9]. Hyposalivation may also lead to difficulties in the formation of food bolus causing pain in swallowing, promoting the development of oral lesions [10].

During anticancer treatments, patients may also develop dysphagia, characterized by pain and swallowing difficulties, whose general prevalence in oncological patients is estimated to be around 15.4% [11]. During RT or combined CT-RT for head and neck cancers (HNC), patients are at increased risk of developing dysphagia, directly related to treatment volumes, thus leading to pain and swallowing difficulties. Dysphagia may be related to changes in neuromusculoskeletal structure and function, anatomical alterations of the head and neck area, saliva alterations, and/or odynophagia [11]. There is a strong relationship between OM, salivary gland hypofunction, and dysphagia.

An additional oral problem is represented by dysgeusia, a taste disorder due to neurological damage, the number of receptor cells, and alterations of the cell structure, in which a persistent gustatory sensation arises in the absence of taste stimulants, or distorted gustatory perception [12]. The gustatory perception could, rarely, be completely (ageusia) or, more often, partially reduced in its intensity (hypogeusia). The prevalence of dysgeusia depends on the cancer treatment: if it is exclusively related to CT, the prevalence is 56.3%; to head and neck RT, it is 66.5%; while with CT-RT, it rises up to 76%. A permanent taste alteration may affect about 15% of the patients [13].

Hyposalivation—OM of the palate and laryngeal tissues, fibrosis, edema and atrophy of vocal folds, and laryngeal/pharyngeal tissues—could lead to voice and/or speech alterations [14]. The pathophysiology of dysphonia may also include neuromuscular weakness due to tumor invasion.

In addition to oral problems related to anticancer therapies, the numb chin syndrome (NCS), a rare form of sensitivity neuropathy manifesting as a sensation of dysaesthesia or anesthesia in the head and neck regions, specifically in the perioral zone [15,16], could be associated to metastatic dissemination rather than CT neurotoxicity itself. This condition is caused by tumoral infiltration or compression of the mandibular nerve or its distal branches like the inferior alveolar nerve or the mental nerve [17]. In most cases, it is due to the presence of bone metastases in the mandibular body, but it can also be caused by metastases of the cranial base or by leptomeningeal dissemination [18]. The typical presentation of NCS is with unilateral sensitivity alterations as dysaesthesia, paraesthesia, or anaesthesia and involves the labial and chin regions [19].

Oral problems could be responsible for dose reduction or discontinuation of anticancer therapies, often associated to patients’ reduced survival rates [19]. Side effects of CT, including oral problems, have been widely collected and analyzed in clinical trials [20]. However, possible biases arising from trials compared to routine care include the exclusion of patients where complications are more expected or a more accurate monitoring of complications. Finally, in clinical trials, oral problems are typically assessed and scored by clinicians, not by patients, often underestimating the number and severity of toxicities experienced by patients [21]. All these considerations lead to the need of collecting observational data in routine clinical practice. Recent data reporting CT-related oral problems examined only specific CT regimens [22,23], tumors [24,25], or toxicities [26,27]. The lack of information from the patients’ perspective on oral problems arising during the routine oncology practice [28], across a range of solid tumors and CT regimens, represented open questions which would be addressed through our prospective observational study.

## 2. Materials and Methods

The prospective observational study was conducted in collaboration between the Department of Oncology (Maggiore Hospital, Trieste, Italy) and the Oral Medicine and Pathology Unit (Maxillofacial Surgery and Odontostomatology Clinic, Maggiore Hospital, Trieste, Italy).

Patients, meeting the following inclusion and exclusion criteria, were consecutively recruited at the day hospital of the Department of Oncology.

Inclusion criteria include the following:

- Male and female genders;
- Age ≥ 18 years;
- Diagnosis of any solid tumor;
- Outpatients receiving second or subsequent CT cycle;
- Signature of written informed consent.

Exclusion criteria include the following:

- Age < 18 years;
- Haematological cancer;
- Hospitalized patients;
- Patients at first CT infusion or not currently receiving CT;
- Concomitant or previous head and neck RT;
- Patients that refused to sign the written informed consent;
- Patients incapable of understanding and/or wanting.

A specific and anonymous questionnaire in the Italian language, validated by the regional ethics committee, was elaborated by our multidisciplinary team (Annex S1). For the entire duration of the study (1-year observation) and for each patient treated with CT in the observation period, the questionnaire was completed during each CT cycle, excluding patients undergoing the first CT cycle.

The questionnaire collected patients’ general information and medical history such as age, gender, tumor diagnosis and stage, presence of metastasis, CT agent type (antimetabolites, mitotic inhibitors, topoisomerase II inhibitors, platinum agents, anti-tumor antibiotics, alkylating agents, multiple CT-based combination), and number of CT cycles.

The questionnaire was planned in relation to the criteria for patients’ subjective assessment and perception of oral problems, in agreement with the MASCC/ISOO evidence-based guidance [2]. Specifically, through the administration of the questionnaire, the following items were assessed: presence of oral and perioral pain, OM, salivary gland hypofunction, dysgeusia, dysphagia, dysphonia, and sensitivity neuropathy (paraesthesia or dysaesthesia). Patients were also asked about frequency and duration, since last CT infusion, of the reported oral problems and about their intensity using the numerical rating scale—an 11 pt numerical scale with boxes (0–10) for each response—since this format proved to have good feasibility, reliability, and convergent validity [29,30].

When patients reported in the questionnaire the onset of oral problems, we investigated which types of medications have been used and who recommended them. Eventually, we investigated the degree of awareness of the patients about the possibility of oral problems arising during CT.

### 2.1. Questionnaire Face and Content Validity

The face validity of the questionnaire, including questions appropriateness, logical sequence, and comprehensibility was administered and examined by 10 patients before the beginning of the study. The impact score (IS) of each item was calculated using a five-point Likert appropriateness scale ranging from 1 (not appropriate at all) to 5 (highly appropriate) and items scoring < 1.5 were removed from the questionnaire [31]. Moreover, content validity was assessed using the content validity ratio (CVR) and content validity index (CVI). Items were classified as not necessary, useful but not essential, or essential, and CVR values, according to Lawshe’s formula [32], lower than 0.62 were removed from the questionnaire. The relevance of each item was defined according to a four-point Likert scale and items with CVI < 0.80 were removed from the final questionnaire [33].

### 2.2. Statistical Analysis

IBM SPSS software version 29.0 for Windows (Armonk, NY, USA) was used to perform the statistical analyses.

Patients’ demographic and medical information were summarized by calculating descriptive statistics, comprising means, standard deviations (SDs), medians, and interquartile ranges (IQRs). After assessing the normality of distribution of data using the Kolmogorov–Smirnov test, a Student’s *t*-test was employed to evaluate any significant differences in patients’ age distribution. The chi-squared test was used to analyze the significance of the differences in categorical variables (tumor diagnosis, presence of metastasis, CT agent type, and number of CT cycles) between genders.

Then, a multiple backward logistic regression model, using backward variable selection, was conducted exploring the considered variables as predictors for oral problems. In particular, the onset of oral problems was considered as the dependent variables (oral and perioral pain, OM, salivary gland hypofunction, dysgeusia, dysphagia, dysphonia, sensitivity neuropathy), while the explanatory variables (categories) entered in each model were age, gender, tumor diagnosis, presence of metastasis, CT agent type, number of CT cycle, and medications to manage oral problems. The cut-off levels of significance were 0.05 and 0.10 for entry and removal, respectively. For each significant association of variables, odds ratios (ORs) and 95% confidence intervals (CIs) were calculated. A *p*-value < 0.05 was considered statistically significant.

## 3. Results

### 3.1. Descriptive Analysis

A total of 194 patients were recruited, 89 males and 105 females. Female mean age was 63.5 ± 11.2, median age 65 (range 32–85), while males mean age was 67 ± 11 years, median age 69 (range 26–83). Any statistically significant difference between groups’ age were found.

A total of 491 questionnaires were collected, with an average of 2.5 questionnaires per person. Female patients filled in 55.6% of the questionnaires, while male patients filled in 44.4%.

Table 1 shows the prevalence of tumor diagnosis and stage, metastasis, CT agent type, and number of CT cycles according to patients’ gender distribution.

The 80% of enrolled patients were informed by the oncologist about possible oral problems arising during CT. We considered the answer given at the first questionnaire compilation, in case the patient filled in more than one questionnaire.

### 3.2. Oral Problems

Patients’ oral problems and their frequency over the last month are presented in Table 2 and Table 3, respectively. Among the considered oral problems, none led to the necessity of suspending, delaying, or modifying the oncological treatment schedules.

Where assessable, the intensities of the oral problems referred to by patients have been assessed through the NRS. Results are summarized in Table 4. Any statistically significant differences among intensity of referred the oral problems were found.

The presence of paraestheria and/or anesthesia in labial or chin regions was reported in 17 questionnaires. Specifically, these patients were undergoing the following antiblastic treatments: one patient, platinum agents; seven patients, antimetabolites; three patients, mitotic inhibitors; and six patients, multiple CT-based combinations.

Anaesthesia was highlighted in 12 questionnaires in patients treated with platinum agents, in 6 questionnaires in patients treated with antimetabolites, and in 2 questionnaires in patients treated with mitotic inhibitors. Moreover, in 12 out 53 questionnaires (22.6%) patients reported the presence of both paraesthesia and anaesthesia.

When oral problems were reported, patients were asked whether any medication has been used and who recommended it for local recovery (Table 5).

With regard to the type of medication used as a symptomatic approach, 277 questionnaires were considered: in 98 questionnaires (35.4%), the treatment was suggested by the oncologist and/or by the nurse staff; in 38 questionnaires (13.7%), by the general practitioner and/or by the dentist; in 27 questionnaires (9.7%), by other health care figures; and 114 questionnaires (41.2%) consisted of homemade treatments. In this latter category, medications were self-prescribed (97/114 patients), prescribed by pharmacists or herbalists (5/114 patients), or suggested by relatives, friends, or other patients (12/114 patients).

### 3.3. Multivariate Analysis

In the multivariate analysis, 481 questionnaires out 491 were included, since 10 patients did not specify their tumor stage in the questionnaire; therefore, this missing data could have generated bias in the data analysis. Multiple backward logistic regression models were performed to correlate the independent variables (age, gender, tumor diagnosis, metastasis, CT agent type, CT cycle, and use of medications to manage oral problems) and oral problems considered in the study (Table 6).

Specifically, OM onset was significantly related to the presence of metastasis (OR = 2.02, *p* = 0.026) and led to the use of medications for his management (one class OR = 3.27, *p* = 0.030; two or more classes OR = 3.42, *p* = 0.017). A salivary gland hypofunction, referred by patients after CT, was more evident in those affected by breast and prostate tumors (OR = 2.68, *p* = 0.015) undergoing platinum agents (OR = 2.16, *p* = 0.013) or multiple CT-based combinations (OR = 2.14, *p* = 0.035). Similarly, most patients submitted to platinum agents or antimetabolites experienced dysphagia (OR = 3.26, *p* = 0.001 and OR = 2.95, *p* = 0.006, respectively). Despite the presence of swallowing difficulties, patients were less prone to take two or more classes of medications (OR = 0.51, *p* = 0.033). Antimetabolites lead to a slight increase in the presence of dysphonia (OR = 1.12, *p* = 0.016), which increased with the number of CT cycles performed (OR = 1.06, *p* = 0.041). However, patients with dysphonia and with labial pain were less prone to use one class of medication to manage this oral problem (OR = 0.45, *p* = 0.022 and OR = 0.48, *p* = 0.026, respectively). The presence of labial pain was less related to male gender (OR = 0.50, *p* = 0.001) and mainly to the presence of metastasis (OR = 1.64, *p* = 0.021). Patients that most referred the presence of paresthesia and anesthesia were treated with multiple CT-based combinations (OR = 6.55, *p* = 0.013) and platinum agents (OR = 5.16, *p* = 0.041), respectively. As the number of CT cycles increased, a slight increase in the occurrence of anesthesia was reported by patients (OR = 1.10, *p* = 0.049).

## 4. Discussion

In the present study, we investigated the presence of oral problems arising after CT regimens in patients affected by solid tumors. Differently from the majority of clinical trials, where oral problems have been recorded by healthcare professionals [21,22], we focused on the impact of these complications from patients’ perspective through the administration of a dedicated questionnaire.

Most of the oral problems complained by oncological patients undergoing CT are related to drug stomatotoxicity [3,4,5,34], as oral pain, OM, salivary gland hypofunction, dysphagia, dysphonia, labial pain, or, in less cases, to tumor metastatic dissemination (NCS, 17).

Among the considered oral problems, OM and its oral manifestations can significantly impact on anticancer therapies, as they can lead to the necessity of suspending, delaying, or modifying the oncological treatment schedules. OM can be associated to oral pain, oral ulcers, dysphagia, and dysphonia. The prevalence of OM and oral pain in our survey was 15.5% (76 questionnaires) and 21.6% (106 questionnaires), respectively. Compared to the literature data, our study seems slightly to underestimate the prevalence of OM. This is likely related to an evaluation only reported by patients and not confirmed by any clinical assessment, unlike of what has been recorded in studies considering the same oral problem [35,36].

It is known that oral pain is not always proportionally related to the presence of OM, but it can be also due to the presence of oral oedema [7]. OM can be very disabling for patients (NRS ≥ 7 in 55.3% of enrolled patients), but it is not necessarily present after every CT cycle, in fact OM was described as sporadic (less than one episode a month) in 30.3% of the questionnaires. In 18–40% of the cases, OM manifests after the first infusional therapy, but sometimes it appears later due to the cumulative effect of some CT regimens and the concomitant CT-induced neutropenia [1]. However, in our analysis, the number of CT cycles did not statistically influence the development of oral pain and OM, like in the cross-sectional study by Wilberg et al. [37]. The presence of oral pain and OM was mostly reported by female (63.2% female vs. 36.8% men for oral pain and 68.4% vs. 31.65%, *p* = 0.014, for OM). However, according to the results of the multivariate analysis, the female gender was not considered a risk factor for the development of oral pain and OM, similarly to the study by Wilberg et al. [37]. On the contrary, the randomized multicentre study by Vukurka et al. showed an increased risk of OM with more severe lesions in female, despite they enrolled only oncohaematological patients undergoing CT before bone marrow transplantation [38]. We also observed that age was a protective factor for the development of both oral pain and OM (OR 0.98, *p* = 0.014 for oral pain; OR 0.95, *p* = 0.000 for OM). The same results were found in relation to salivary gland hypofunction (0.98, *p* = 0.013) and labial pain (OR 0.98, *p* = 0.009). These data are in disagreement with the literature where OM is usually more frequent and more severe in people older than 50 years, probably for the concomitant renal failure and the consequent CT dose accumulation [7]. We did not find a correlation between the development of oral pain and OM and CT agent type, probably because in each category there were stomatotoxic drugs. Unfortunately, any other observational study considered this risk factor [39,40] or, conversely, were considered only specific schemes or restricted number of molecules [41,42,43]. On the contrary, in our study, the presence of metastatic disease was a risk factor for the development of OM (OR 2.02, *p* = 0.026) and salivary gland hypofunction (OR 1.66, *p* = 0.042), likely related to a worse performance status and more aggressive CT regimens.

Alteration in salivary production was the most referred symptom in our study group (44.2%). Similar results were obtained in the cross-sectional study from Frowen et al. [44], where the prevalence of patient-reported xerostomia was 56%. Despite hyposalivation being referred as always present in 53.5% of the questionnaires, it did not cause a great discomfort (mean NRS = 4.9), similarly to the results obtained by Mercadante et al. [39] and Chen et al. [40]. A correlation was found between these manifestations and the female gender, to note 60.4% of questionnaires concerning salivary gland hypofunction were submitted by females, despite with no statistically significant differences. The administration of platinum agents and multiple CT-based combination represented a risk factor for the development of salivary gland hypofunction compared to mitotic inhibitors and antimetabolites (OR = 2.16, *p* = 0.013 and OR = 2.14, *p* = 0.035, respectively).

Furthermore, we found a significative correlation between the presence of dysphagia and the use of platinum agents and antimetabolites compared to mitotic inhibitors (OR 3.26, *p* = 0.001 and OR 2.95, *p* = 0.006, respectively). It is interesting to observe that 47 questionnaires complaining about dysphagia were all related to therapy containing platinum agents and that platinum agents were the most frequent category related to dysphagia for solids (9 out 18), liquids (11 out 19), and both (27 out 57). This drug can cause stomatotoxicity but also neurotoxicity, with dysphagia and pharyngolaryngeal dysaesthesia, mostly related to low temperature exposition, as drinking cold water. In our study, dysphagia for liquids was platinum-related in 11 questionnaires out of 19. However, we did not investigate the cold correlation due to the small sample of questionnaires complaining about this disorder, compared to the prospective, multicenter, international study of Argyriou et al., in which the cold-related pharyngolaryngeal dysaesthesia reached 91.8% of patients receiving oxaliplatinum [45]. Dysphagia was reported by patients as an intermittent symptom, occurring once or twice a month. Dysphagia was referred as very intense (46.8% questionnaires with NRS ≥ 7) or as a slight disturbance (36.0% questionnaires with NRS ≤ 4), with an average of 5.9. Similar results were reported by Mercadante et al., where 15% of patients reported dysphagia, with an average intensity of 5.3 related to the NRS [39]. Even Chen et al. found that the difficulty in swallowing in CT-treated patients was a disorder mostly referred as mild or moderate [40].

The frequency of dysphonia in our study (32.4% of all questionnaires) was very similar to the one found by Chen et al. (31.5%), even if their sample was smaller [40], and by Frowen et al. [44]. Even in the study from Chen et al., the lowering of vocal tonality was greater than pain in phonation (83.3% vs. 16.7%, 40). The use of antimetabolites increased the risk of this disorder compared to mitotic inhibitors (OR 1.12, *p* = 0.016), probably for the inflammation and oedema of both oral and upper airways mucosa.

Besides oral problems in cancer patients undergoing CT for solid tumors, a dedicated attention has been posed to the NCS, a rare sensitivity neuropathy that usually expresses itself with unilateral sensitivity alterations in lip and/or chin regions. It is mostly caused by bone metastases in the mandibular body, which represents less than 1% of all bone metastases [46] and its onset seems to be a negative prognostic factor for patient’s survival [47].

The presence of paraesthesia and total anaesthesia in these regions were referred to, respectively, in 33 and 20 questionnaires. Among these, 12 patients referred to a difference between the left and right sides, where on the one side reported a reduced sensitivity (paraesthesia) and on the other its complete absence (anaesthesia). Only five patients referred this symptom as unilateral, so we investigated this symptom in relation to their medical history in order to consider the diagnosis of NCS. However, in none of them NCS was confirmed by instrumental diagnostic examinations (computed tomography, nuclear magnetic resonance, PET-CT, bone scintigraphy). The absence of diagnosis of NCS is probably due to the small sample size of patients with bone metastases 26 (13.6%).

A great part of questionnaires (29 out 53), reporting paraesthesia and/or anaesthesia, referred to the use of platinum agents, which are known for causing acute and chronic neurotoxicity involving also the perioral area [48]. In addition, 15 out 24 of non-platinum-related questionnaires came from patients with previous platinum CT scheme. The statistical analysis showed a significative increased risk of anaesthesia in patients undergoing platinum agents compared to mitotic inhibitors (OR 5.16, *p* = 0.041). We also observed a little increased risk of anaesthesia related to the number of CT cycles, probably due to its cumulative effect (OR 1.10, *p* = 0.049). Even the prospective study of Argyriou et al. showed a correlation between the use of platinum agents and the development of perioral paraesthesia: 95.2% of patients complained about this cold-related disorder [45]. However, their high incidence could be due to the exclusive enrollment of patients undergoing oxaliplatinum therapy, particularly neurotoxic [49]. On the contrary, Rahnma et al., in a case-control study, considered different CT regimens (oxaliplatinum, cisplatin, doxorubicin, fluorouracil, docetaxel, and cyclophosphamide) and found the presence of tingling and numbness in the inferior lip in 22.4% patients [50]. Our results highlighted that the multiple CT-based combination group increased the risk of perioral paraesthesia compared to mitotic inhibitors (OR 6.55, *p* = 0.013), probably for the large presence of vinca alkaloids in this category, known for causing neurotoxicity [37]. Moreover, the perioral paraesthesia and/or anaesthesia were referred as present once or twice a month in the 63% of the questionnaires, probably related to the infusional CT scheme performed (every 14, 21, or 28 days).

To manage these oral problems, in several questionnaires, patients reported the employment of medications. This is reasonable if we consider that in 138 questionnaires, no symptoms were referred to, while in many cases, symptoms were modest or resolved in a short time. On the contrary, in 96 questionnaires, it was reported that the use of bicarbonate rinses and/or an antimicotic was routinely recommended by the medical and nursing staff in our university hospital.

Some interesting data concerns the number of patients (20%) who declared that did they not receive information about the possibility of an onset of oral problems during CT and their management. However, our results are much more encouraging than those reported in the literature. Wilberg et al. showed that 73% of the patients enrolled were unprepared about possible oral CT-related side effects [37].

Differently from other similar studies [20,21], where oral problems are typically assessed by clinicians, often underestimating the number and severity of toxicities experienced by patients, the present study analyzed the patients’ perspective. The obtained results highlighted the importance of collecting observational data from the patients’ perspective on oral problems arising during the routine oncology practice, across a range of solid tumors and CT regimens. Our data provide guidance for planning an evidence-based framework for the management of oral problems in oncological patients, although every patient requires individualized management. This could include the planning of patient-oriented training sessions (through brochures, videos, or apps). Indeed, the relevance of our findings focused on the key role of the multidisciplinary team in advising the patients on the possible occurrence of oral problems, also by recommending their management.

Alongside the clinical relevance of our study, some limitations need to be discussed. A heterogeneous sample of patients was enrolled, with different ages, tumors diagnoses (according to site and staging), and consequently different types of CT schemes. Similarly, patient’s socio-demographic characteristics and local and general conditions could be implemented (i.e., considering patients’ race, social education, body mass and Karnofsky indexes, pre-existing co-morbidities, oral hygiene, periodontal disease, etc.).

While patients’ self-reported oral problems represent an unquestionably strong point of the study, which distinguishes it from already published studies on the same topic, a clinical evaluation could be useful to compare subjective and objective differences in the clinical manifestation of oral problems CT-related. Furthermore, an increase in patients’ sample is essential to identify the NCS, particularly including patients with bone metastases over a longer follow up.

## 5. Conclusions

The results of the present study, reporting the patients’ perspective on oral problems arising during anticancer therapies, underline the importance of monitoring subjective symptoms during the clinical routine. The relevance of our findings focused on the key role of the multidisciplinary team in advising patients on the possible occurrence of oral problems, also by preventing their onset or recommending their management through awareness campaigns. Future research should assess the effect of self-reporting oral problems on clinical outcomes and the efficiency for early detection of adverse events related to anticancer therapies.

## Figures and Tables

**Table 1:** Distribution of tumor diagnosis and stage, presence of metastasis, CT agent type, and number of CT cycles according to gender (chi-squared Test). ** Significance *p* ≤ 0.01. NS—not significative.

	Female			Male		Diff.
	Frequency (Total 105)		Percentage (Total 100%)	Frequency (Total 89)	Percentage (Total 100%)	
**Tumor diagnosis**						*p* < 0.0001 **
Lung and pleura	13		12.4%	32	36.0%	
Gastrointestinal	30		28.6%	35	39.3%	
Pancreas and hepatobiliary	12		11.4%	10	11.2%	
Prostate	0		0.0%	1	1.1%	
Breast	28		26.7%	0	0.0%	
Ovarian	13		12.4%	0	0.0%	
Uterus	3		2.9%	0	0.0%	
Testis	0		0.0%	4	4.5%	
Kidney and urinary tract	2		1.9%	2	2.3%	
Melanoma	0		0.0%	2	2.3%	
Soft tissue	1		0.9%	1	1.1%	
Central nervous system	1		0.9%	1	1.1%	
Unknown origin	2		1.9%	1	1.1%	
**Tumor stage**						NS
Stage I	5		4.76%	6	6.74%	
Stage II	7		6.67%	8	8.99%	
Stage IIII	24		22.86%	23	25.84%	
Stage IV	69		65.71%	52	58.43%	
**Metastasis**						NS
Yes	69	65.7%	52	58.4%	69	
No	36	34.3%	37	41.6%	36	
**CT agent type**						NS
Antimetabolites	17	16.2%	9	10.1%	17	
Mitotic inhibitors	18	17.1%	7	7.9%	18	
Topoisomerase II inhibitors	1	0.95%	0	0.0%	1	
Platinum agents	4	3.8%	0	0.0%	4	
Anti-tumor antibiotics	2	1.9%	1	1.1%	2	
Alkylating agents	1	0.95%	2	2.2%	1	
Multiple CT-based combination	62	59.1%	70	78.7%	62	
**CT cycle**						NS
Equal or less than 5	84	80%	70	78.7%	84	
More than 5	21	20%	19	21.3%	21	

**Table 2:** Frequencies of answers to the questionnaire and distribution of answers according to gender (Pearson chi-square test). * Significance 0.01 < *p* ≤ 0.05. ** Significance *p* ≤ 0.01. NS—not significative.

		Overall Sample			Female		Male		Diff.
		Frequency (Total 491)		Percentage (Total 100%)	Frequency (Total 273)	Percentage (Total 100%)	Frequency (Total 218)	Percentage (Total 100%)	
**Oral pain**	**No**	385		78.4%	206	53.5%	179	46.5%	NS
	**Yes**	106		21.6%	67	63.2%	38	36.8%	
								
**Oral mucositis**	**No**	415		84.5%	221	53.3%	194	46.7%	*p* = 0.014 *
	**Yes**	76		15.5%	52	68.4%	24	31.6%	
								
**Salivary gland hypofunction**	**No**	274		55.8%	142	51.8%	132	48.2%	NS
	**Yes**	217		44.2%	131	60.4%	86	39.6%	
								
**Dysphagia**	**No**	397		80.9%	224	56.4%	173	43.6%	NS
	**Yes**	94		19.1%	49	52.1%	45	47.9%	
		For solids	18	19.2%	8	44.4%	10	55.6%	
		For liquids	19	20.2%	9	47.4%	10	52.6%	
		For both	57	60.6%	32	56.1%	25	43.9%	
								
**Dysphonia**	**No**	332		67.6%	189	56.9%	143	43.1%	NS
	**Yes**	159		32.4%	84	52.8%	75	47.2%	
		Low and/or hoarse voice	139	87.4%	68	48.9%	71	51.1%	
		Pain during phonation	20	12.6%	16	80.0%	4	20.0%	
								
**Labial pain**	**No**	306		62.3%	150	49.0%	156	51.0%	*p* = 0.000 **
	**Yes**	185		37.7%	123	66.5%	62	33.5%	
		Dryness	173	93.5%	113	65.3%	60	34.7%	
		Ulcers	12	6.5%	10	83.3%	2	16.7%	
									
**Paraesthesia**	**No**	458		93.3%	255	55.7%	203	44.3%	NS
	**Yes**	33		6.7%	18	54.5%	15	45.5%	
		Unilateral	4	12.1%	1	25.0%	3	75.0%	
		Bilateral	29	87.9%	17	58.6%	12	41.4%	
								
**Anaesthesia**	**No**	471		95.9%	261	55.4%	210	44.6%	NS
	**Yes**	20		4.1%	12	60.0%	8	40.0%	
		Unilateral	5	25.0%	1	20.0%	4	80.0%	
		Bilateral	15	75.0%	11	73.3%	4	26.7%	

**Table 3:** Frequency of oral problems over the last month.

	Oral Pain		Oral Mucositis		Salivary Gland Hypofunction		Dysphagia		Dysphonia		Labial Pain		Paraesthesia and/or Anaesthesia	
	No.	%	No.	%	No.	%	No.	%	No.	%	No.	%	No.	%
**Always** **present**	24	22.6%	18	23.7%	116	53.5%	16	17.0%	46	28.9%	113	61.1%	10	24.4%
**3 weeks**	3	2.8%	0	0.0%	7	3.2%	0	0.0%	6	3.8%	3	1.6%	1	2.4%
**2 weeks**	1	0.9%	1	1.3%	26	12.0%	5	5.3%	25	15.7%	14	7.6%	3	7.3%
**1 week**	4	3.8%	3	3.9%	10	4.6%	7	7.45%	13	8.2%	10	5.4%	0	0.0%
**Twice/month**	22	20.8%	7	9.2%	25	11.5%	23	24.5%	35	22.0%	15	8.1%	5	12.2%
**Once/month**	40	37.7%	24	31.6%	30	13.8%	36	38.3%	28	17.6%	21	11.3%	18	43.9%
**Rarely**	12	11.4%	23	30.3%	3	1.4%	7	7.45%	6	3.8%	9	4.9%	4	9.8%
Total	106	100%	76	100%	217	100%	94	100%	159	100%	185	100%	41	100%

**Table 4:** Intensity of oral problems (11 pt numerical rating scale from 0 to 10).

	NRS Oral Pain		NRS Oral Mucositis		NRS Salivary Gland Hypofunction		NRS Dysphagia		NRS Paraesthesia and/or Anaesthesia	
	Frequency	Percentage	Frequency	Percentage	Frequency	Percentage	Frequency	Percentage	Frequency	Percentage
**0**	0	0.0%	0	0.0%	0	0.0%	0	0.0%	0	0.0%
**1**	1	0.9%	0	0.0%	13	6.0%	0	0.0%	1	24%
**2**	8	7.5%	3	3.9%	22	10.1%	6	6.4%	5	12.2%
**3**	9	8.5%	7	9.2%	27	12.4%	12	12.8%	8	19.5%
**4**	10	9.4%	7	9.2%	27	12.4%	16	17.0%	8	19.5%
**5**	24	22.6%	12	15.8%	53	24.4%	7	7.4%	4	9.8%
**6**	11	10.4%	5	6.6%	20	9.3%	9	9.6%	6	14.6%
**7**	13	12.4%	15	19.7%	20	9.3%	16	17.0%	5	12.2%
**8**	18	17.0%	13	17.2%	23	10.6%	15	16.0%	2	4.9%
**9**	5	4.7%	3	3.9%	2	0.9%	7	7.4%	2	4.9%
**10**	7	6.6%	11	14.5%	10	4.6%	0	0.0%	0	0.0%
Average	5.8		6.5		4.9		5.9		4.7	

**Table 5:** Medications to manage oral problems.

	Frequency (Total 491)		Percentage (Total 100%)
**Total n. medications**	214		43.6%
**Bicarbonate and/or antifungal rinses**	96		19.5%
**Others**	181		36.9%
	Mouth and/or labial hydration	59	32.6%
	Mouthrinses (not specified)	57	31.5%
	Mouthrinses with chlorhexidine	27	14.9%
	Natural local products	17	9.4%
	Specific products for oral mucositis	10	5.5%
	Photobiomodulation	5	2.8%
	Gel	2	1.1%
	Topical antiviral	2	1.1%
	Unknown	2	1.1%
		Total 181	Total 100%

**Table 6:** Multiple backward logistic regression model predicting oral problems in cancer patients. OR—odds ratio; CI—confidence interval. * Significance 0.01 < *p* ≤ 0.05. ** Significance *p* ≤ 0.01. NS—not significative.

**Independent Variables**		**Oral Pain**		**Oral Mucositis**		**Salivary Gland Hypofunction**		**Dysphagia**		
	**No.**	**OR (95% CI)**	**Diff.**	**OR (95% CI)**	**Diff.**	**OR (95% CI)**	**Diff.**	**OR (95% CI)**	**Diff.**	
**Age**		0.98 (0.96–1.00)	0.014 **	0.95 (0.93–0.97)	0.000 **	0.98 (0.96–1.00)	0.013 *	0.96 (0.94–0.98)	0.000 **	
**Gender**		NS	NS			NS	NS	NS	NS	
Female	263				1					
Male	218			0.59 (0.34–1.03)	NS					
**Tumor diagnosis**		NS	NS	NS	NS		0.016 *	NS	NS	
Pancreas and hepatobiliary	60						1			
Miscellaneous	78					0.87 (0.42–1.79)	NS			
Breast and prostate	91					2.68 (1.22–5.90)	0.015 *			
Lung and pleura	83					0.82 (0.39–1.71)	NS			
Gastrointestinal	169					0.96 (0.48–1.89)	NS			
**Metastases**		NS	NS					NS	NS	
No	169				1		1			
Yes	312			2.02 (1.09–3.74)	0.026 *	1.66 (1.02–2.70)	0.042 *			
**CT agent type**		NS	NS	NS	NS		0.017 *		0.008 **	
Mitotic inhibitors	116						1		1	
Platinum agents	198					2.16 (1.17–3.96)	0.013 *	3.26 (1.62–6.56)	0.001 **	
Antimetabolites	97					0.98 (0.48–2.00)	NS	2.95 (1.36–6.44)	0.006 **	
Multiple CT-based combination	70					2.14 (1.06–4.33)	0.035 *	1.97 (0.81–4.78)	NS	
**CT cycle**		NS	NS	NS	NS	0.95 (0.89–1.00)	NS	NS	NS	
**Medications**		NS	NS		NS		0.016 *		NS	
None	74				1		1		1	
One class	104			3.27 (1.13–9.49)	0.030 *	0.60 (0.31–1.15)	NS	0.53 (0.26–1.10)	NS	
Two or more classes	303			3.42 (1.25–9.35)	0.017 *	1.25 (0.70–2.23)	NS	0.51 (0.28–0.95)	0.033 *	
**Independent Variables**		**Dysphonia**		**Labial Pain**		**Paresthesia**		**Anaesthesia**		
	**No.**	**OR (95% CI)**	**Diff.**	**OR (95% CI)**	**Diff.**	**OR (95% CI)**	**Diff.**	**OR (95% CI)**		**Diff.**
**Age**		NS	NS	0.98 (0.96–0.99)	0.009 **	0.97 (0.94–1.00)	NS	0.93 (0.90–0.97)		0.000 **
**Gender**		NS	NS			NS	NS	NS		NS
Female	263				1					
Male	218			0.50 (0.34–0.74)	0.001 **					
**Tumor diagnosis**		NS	NS	NS	NS		0.021 *	NS		NS
Pancreas and hepatobiliary	60						1			
Miscellaneous	78					0.11 (0.01–0.98)	0.048 *			
Breast and prostate	91					0.76 (0.18–3.18)	NS			
Lung and pleura	83					0.30 (0.07–1.33)	NS			
Gastrointestinal	169					1.99 (0.63–6.32)	NS			
**Metastases**		NS	NS			NS	NS	NS		NS
No	169				1					
Yes	312			1.64 (1.08–2.49)	0.021 *					
**CT agent type**			0.046 *	NS	NS		0.027 *			NS
Mitotic inhibitors	116		1				1			1
Platinum agents	198	1.06 (0.63–1.79)	NS			3.50 (0.83–14.79)	NS	5.16 (1.07–24.88)		0.041 *
Antimetabolites	97	1.12 (1.15–3.90)	0.016 *			1.38 (0.27–6.95)	NS	2.70 (0.41–17.71)		NS
Multiple CT-based combination	70	1.48 (0.76–2.89)	NS			6.55 (1.48–29.07)	0.013 *	NS		NS
**CT cycle**		1.06 (1.00–1.11)	0.041 *	NS	NS	NS	NS	1.10 (1.00–1.22)		0.049 *
**Medications**			0.046 *		0.020 *	NS	NS	NS		NS
None	74		1	1	1					
One class	104	0.45 (0.23–0.89)	0.022 *	0.48 (0.25–0.92)	0.026 *					
Two or more classes	303	0.80 (0.46–1.4)	NS	0.97 (0.56–1.68)	NS					

## Data Availability

Data available on request due to privacy restrictions. The data presented in this study are available on request from the corresponding author.

## Supplementary Materials

The following supporting information can be downloaded at: https://www.mdpi.com/article/10.3390/cancers16010176/s1, Annex S1: Questionnaire on Oral Problems in Oncology Patients Undergoing Chemotherapy for Solid Tumors.
