# Supplementary material for: Oral Problems in Oncology Patients Undergoing Chemotherapy for Solid Tumors: A Prospective Observational Study
Source: Cancers (Basel). 2023 Dec 29;16(1):176. doi: 10.3390/cancers16010176 (PMC10778343; doi:10.3390/cancers16010176)
Supplement: Supplementary file 1 [file cancers-16-00176-s001.zip › cancers-2746703-supplementary.pdf]

Supplementary Materials. Questionnaire on oral problems in oncology patients undergoing chemotherapy for solid tumors.

Patient ID (compiled by operators): ______________________

Compilation date (compiled by operators): ___ /___ /____

Patient's general and medical information

Please select your gender ☐ Male ☐ Female ☐ I prefer not to answer

Please report your date of birth: ____________

Select your tumor diagnosis:

☐ Lung and pleura

☐ Gastrointestinal

☐ Pancreas and hepatobiliary

☐ Prostate

☐ Breast

☐ Ovarian

☐ Uterus

☐ Testis

☐ Kidney and urinary tract

☐ Melanoma

☐ Soft tissue

☐ Central nervous system

☐ Unknown origin

Select your tumor stage:

☐ Stage I

☐ Stage II

☐ Stage III

☐ Stage IV

To the best of your knowledge, has the tumour generated metastases (localisation of the tumour to other sites)?

☐ Yes

☐ No

Please report the name of the chemotherapy you are undergoing (this information can be found on the informed consent given by your oncologist):

____

____

This cycle of chemotherapy you are starting today is number ______________

Self-reported oral problems

After the last CT cycle have you ever had:

ORAL PAIN Yes ☐ No ☐

If yes, please tick the maximum intensity of oral pain you experienced:
0 (no pain) 1 2 3 4 5 6 7 8 9 10 (the worst pain you can imagine)
If yes, how many times have you felt oral pain from the last CT cycle?

- ☐ Always present
- ☐ 3 weeks
- ☐ 2 weeks
- ☐ 1 week
- ☐ Twice/month
- ☐ Once/month
- ☐ Rarely

ORAL MUCOSITIS (acute inflammation and/or ulceration of the oral mucosa) ☐ Yes ☐ No

If yes, please tick the maximum intensity of pain related to oral mucositis you experienced:
0 (no pain) 1 2 3 4 5 6 7 8 9 10 (the worst pain you can imagine)
If yes, how many times have you felt pain related to oral mucositis from the last CT cycle?

- ☐ Always present
- ☐ 3 weeks
- ☐ 2 weeks
- ☐ 1 week
- ☐ Twice/month
- ☐ Once/month
- ☐ Rarely

SALIVARY GLAND HYPOFUNCTION (dry, doughy mouth) ☐ Yes ☐ No

If yes, please tick the maximum intensity of discomfort associated to dry/doughy mouth you experienced:
0 (no discomfort) 1 2 3 4 5 6 7 8 9 10 (the worst discomfort you can imagine)
If yes, how many times have you felt discomfort associated to dry/doughy mouth from the last CT cycle?

- ☐ Always present
- ☐ 3 weeks
- ☐ 2 weeks
- ☐ 1 week
- ☐ Twice/month
- ☐ Once/month
- ☐ Rarely

DYSPHAGIA (difficulty in swallowing) Yes ☐ No ☐

If yes, please tick the maximum intensity of pain associated to dysphagia you experienced:
0 (no pain) 1 2 3 4 5 6 7 8 9 10 (the worst pain you can imagine)
If yes, how many times have you felt pain associated to dysphagia from the last CT cycle?

- ☐ Always present
- ☐ 3 weeks
- ☐ 2 weeks
- ☐ 1 week
- ☐ Twice/month
- ☐ Once/month
- ☐ Rarely

DYSPHONIA (voice alteration) Yes ☐ No ☐

If yes, how many times have you felt a voice alteration from the last CT cycle?

- ☐ Always present
- ☐ 3 weeks
- ☐ 2 weeks
- ☐ 1 week
- ☐ Twice/month
- ☐ Once/month
- ☐ Rarely

LABIAL PAIN Yes ☐ No ☐

If yes, please tick the maximum intensity of labial pain you experienced:
0 (no pain) 1 2 3 4 5 6 7 8 9 10 (the worst pain you can imagine)
If yes, how many times have you felt labial pain from the last CT cycle?

- ☐ Always present
- ☐ 3 weeks
- ☐ 2 weeks
- ☐ 1 week
- ☐ Twice/month
- ☐ Once/month
- ☐ Rarely

From the last CT cycle, have you ever felt a reduced sensitivity or a sense of anesthesia (like after dental procedures) in your lip and/or chin? Yes ☐ No ☐

If yes, the sensitivity was reduced (paresthesia) ☐ or completely absent (anesthesia)? ☐

If yes, was one side involved ☐ or both sides? ☐

If yes, please indicate with a cross the area or areas involved by this sensation in the figure below:

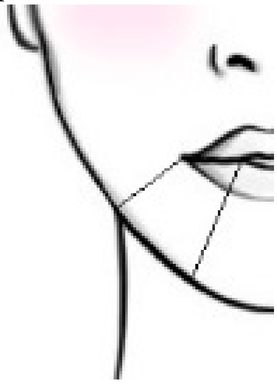

How did you treat these oral problems?

____

____

____

____

Who recommended you these treatments?

☐ Oncologist and Nurse staff
☐ General Practitioner and/or dentist
☐ Other healthcare figures
☐ They are homemade treatment
☐ Other, specify ____________________

If they are homemade treatments, who recommended them to you?

☐ Myself
☐ Pharmacist or herbalist
☐ Relatives, friends, other patents
☐ Other, specify ____________________

Were you aware that oral problems could arise after chemotherapy?

Yes ☐ No ☐
